# Solvent-Free Dual-Curable Waterborne Polyurethane Adhesives Based on Vanillin and Acrylate Monomers

**DOI:** 10.3390/polym18080975

**Published:** 2026-04-17

**Authors:** Weiling Hu, Xiao Zhang, Hao Li, Hengyuan Liang, Can Lin, Zhuo Li, Jia Liu, Feng Feng

**Affiliations:** 1 State Key Laboratory of Green Chemical Synthesis and Conversion, Zhejiang Key Laboratory of Surface and Interface Science and Engineering for Catalysts, Zhejiang University of Technology, Hangzhou 310014, China; 221123010378@zjut.edu.cn; 2 College of Chemical and Material Engineering, Quzhou University, Quzhou 324000, China; zx2915@qzc.edu.cn (X.Z.); lh17738544683@163.com (H.L.); 15925897909@163.com (H.L.); 19557076091@163.com (C.L.); lz20040917@163.com (Z.L.)

**Keywords:** solvent-free waterborne polyurethane, vanillin-derived latent-curing component, dual-curable system, self-emulsification, staged curing behavior

## Abstract

To address the trade-off between storage stability and curing reactivity in NCO-terminated waterborne polyurethane (WPU) systems, a solvent-free WPU emulsion with dual-curing characteristics was developed using vanillin (VAN) and 2-hydroxyethyl acrylate/pentaerythritol triacrylate (HEA/PETA). Hexamethylene diisocyanate (HDI) and 2,2-bis(hydroxymethyl)butyric acid (DMBA) were used as the isocyanate component and internal hydrophilic moiety, respectively, to prepare a self-dispersible polyurethane prepolymer. VAN was introduced as a latent isocyanate-related component, while HEA/PETA served as acrylate-bearing reactive modifiers, followed by self-emulsification to form a stable aqueous dispersion. The prepolymer structure, curing behavior, and adhesive performance on bamboo substrates were systematically investigated. The results supported the successful introduction of VAN-derived structures into the polyurethane chains and the retention of polymerizable C=C bonds from HEA/PETA. Thermal analysis suggested dual-curing behavior with two distinguishable thermal events, involving lower-temperature polymerization of unsaturated groups and a VAN-related higher-temperature reaction. The resulting WPU exhibited dry and wet shear strengths above 23 MPa and 9 MPa, respectively. These findings demonstrate a feasible strategy for integrating emulsion stability, staged curing, and adhesive performance in solvent-free WPU systems.

## 1. Introduction

Waterborne polyurethane (WPU) has been widely used in wood bonding, packaging lamination, and functional coatings because of its adjustable structure and ability to form continuous, dense films [1,2,3]. However, under application conditions that demand high solids content, rapid curing, and long-term service in hot and humid environments, WPU systems derived from NCO-terminated prepolymer routes or containing latent/residual isocyanate functionality may face significant limitations. In such systems, residual or regenerated isocyanate groups can react competitively with water during processing or storage, leading to urea formation, viscosity increase, particle-size instability, and, in some cases, gelation [4]. Furthermore, simply increasing the hard-segment content or introducing multifunctional crosslinking sites during synthesis to improve strength and durability often compromises colloidal stability and narrows the processing window [5,6]. These observations suggest that high-performance WPU design depends not merely on increasing the number of reactive sites, but on coordinated control over latent terminal reactivity and the sequence of network formation during curing [7].

Blocked-isocyanate chemistry offers an important route to efficient, stable, and high-performance WPU bonding under relatively mild conditions [8]. By reacting a blocking agent with –NCO to form a reversible adduct, the system can maintain low reactivity and good storage stability at room temperature. Upon heating, the blocking group is removed, regenerating the –NCO functionality and triggering subsequent crosslinking and curing. The molecular structure of the blocking chemistry directly governs the deblocking temperature, deblocking rate, and the residue behavior of the system after thermal activation [9]. A wide variety of blocking agents have been reported, including oximes, alcohols, phenols, imidazole/pyrazole derivatives, and amides, yet each class presents certain limitations [10,11]. For example, some oxime-based blocking agents may generate low-reactivity cyclized products after deblocking and are often associated with toxicity and odor concerns. Alcohol-based systems generally require relatively high deblocking temperatures. Phenolic blocking agents are strongly affected by steric hindrance, making it difficult to balance blocking and deblocking kinetics, and some low-molecular-weight phenols raise potential safety concerns. Imidazole- and pyrazole-based systems often require higher deblocking temperatures. Although amide-based systems may lower the deblocking temperature through intramolecular hydrogen bonding, they can still suffer from issues related to by-product toxicity and formulation compatibility [12]. These limitations are particularly problematic in waterborne systems, where excessively high deblocking temperatures or significant blocking-agent migration and volatilization may increase energy consumption, leave residual irritating odor, and cause void formation within the adhesive layer [13,14].

Recent studies have shown that bio-based aromatic molecules offer considerable promise for reconciling low-temperature deblocking with sustainability [15]. Among them, vanillin (VAN), which contains a phenolic hydroxyl group, an aldehyde group, and an aromatic ring, can be introduced into polyurethane systems to generate vanillin-derived latent isocyanate-related structures under catalytic conditions, while its aromatic framework may also contribute to increased rigidity of the cured material. It is therefore regarded as a promising low-temperature blocking monomer [13,16,17]. Nevertheless, if crosslinking in WPU systems relies solely on the small amount of –NCO groups regenerated upon deblocking to form a polyurethane network, the adhesive layer may still suffer from insufficient crosslink density and network heterogeneity under conditions of abundant moisture or limited availability of effective reactive components, resulting in locally inadequate cohesive strength [8,18]. Introducing acrylate double bonds as additional curing sites can provide a second network through free-radical polymerization during curing, thereby shifting the development of a highly crosslinked structure from prepolymer preparation to the final curing stage [19,20]. The resulting co-crosslinked or semi-interpenetrating network structure can improve adhesive-layer modulus, energy dissipation, and resistance to medium penetration [21,22]. Overall, previous studies have focused mainly on screening individual blocking agents or enhancing the performance of WPUA systems, whereas systematic investigation of a cooperative dual-blocking/dual-curing mechanism remains limited [22,23,24].

When introduced into polyurethane systems as functional modifiers, 2-hydroxyethyl acrylate (HEA) and pentaerythritol triacrylate (PETA) can provide acrylate-bearing reactive functionalities that regulate terminal-group chemistry and supply polymerizable C=C bonds for subsequent curing [25,26]. Incorporation of HEA/PETA into a WPU system embeds polymerizable unsaturated units within the polyurethane chains, enabling rapid crosslinking during subsequent curing and potentially improving adhesive-layer hardness, adhesion, and water resistance [23,25,27]. On this basis, the present study proposes a vanillin/acrylate combined design to construct a dual-functional and dual-curable system. After formation of the NCO-terminated prepolymer, Vanillin was introduced as the latent isocyanate-related component, whereas HEA/PETA were incorporated as acrylate-bearing reactive modifiers to form the modified prepolymer. Combined with a self-emulsification route, this design enabled the preparation of a solvent-free WPU adhesive. Using hexamethylene diisocyanate (HDI) as the isocyanate source, a difunctional isocyanate-terminated prepolymer containing a hydrophilic center was first constructed with DMBA, followed by chain extension with a mixed polyol system. The residual isocyanate groups were then cooperatively blocked with VAN and HEA/PETA to form a dual-blocked prepolymer. After neutralization with triethylamine and self-emulsification, a stable WPU dispersion was obtained. This work systematically examines how the dual-blocked structure regulates emulsion stability, thermal-curing behavior, adhesive performance, and hydrothermal resistance, with the aim of elucidating the structure–property relationship and providing both theoretical guidance and experimental support for the development of environmentally friendly, high-performance WPU adhesives.

## 2. Materials and Methods

### 2.1. Materials

The main chemicals used in this study included hexamethylene diisocyanate (HDI), vanillin (VAN), 2-hydroxyethyl acrylate (HEA), pentaerythritol triacrylate (PETA), 2,2-bis(hydroxymethyl)butyric acid (DMBA), dibutyltin dilaurate (DBTDL), triethylamine (TEA), and potassium hydroxide (KOH). All were of analytical grade (AR) and purchased from Shanghai Aladdin Biochemical Technology Co., Ltd. (Shanghai, China) without further purification. The macropolyols used were polycarbonate diol (PCDL, Mn = 500), polyethylene glycol (PEG, Mn = 400), and polypropylene glycol (PPG, Mn = 400), also purchased from Shanghai Aladdin Biochemical Technology Co., Ltd., and used directly in the specified proportions. Deionized water was prepared in-house, with a resistivity of approximately 16.2 MΩ·cm.

### 2.2. Preparation of Waterborne Polyurethane

A mixture of 8.0 g of DMBA and 50.46 g of HDI was charged into a reaction flask pre-purged with argon, followed by the addition of DBTDL at 0.15 wt% of the total formulation as a catalyst. The reaction was carried out at 80 °C and 300 r/min for 50 min to obtain Prepolymer 1. After Prepolymer 1 had formed, the system was cooled to 65 °C, and a mixed polyol solution was slowly added dropwise by peristaltic pump at 0.6 mL/min, with a molar ratio of PCDL:PPG:PEG = 4:4.2:1.8. The reaction was then continued for 90 min to yield Prepolymer 2. The temperature was subsequently lowered to 60 °C. According to the formulation listed in Table 1, VAN, HEA, and PETA were weighed, premixed in a sealed vessel at room temperature (20–30 °C) under stirring until a homogeneous mixture was obtained, and then added to the reactor as a single feed. The total molar feed ratio of the VAN/HEA/PETA mixture to the residual –NCO groups was fixed at 0.9:1. The reaction was then allowed to proceed for a further 90 min to give the modified prepolymer. After that, 4.64 g of TEA was added for neutralization, and the mixture was maintained for 30 min. The amount of TEA used for neutralization was calculated from the carboxylic acid equivalents of DMBA and corresponded to 85% neutralization. For 8.0 g of DMBA (98% purity, M = 148.16 g mol^−1^), the required TEA amount was calculated to be 4.64 g (TEA purity 98%, M = 101.19 g mol^−1^). Finally, 0.45 g of KOH was dissolved in 342 g of deionized water, and this aqueous solution was added dropwise into the system at 10 mL/min under stirring. The stirring speed was increased to 1000 r/min, and emulsification was continued for 15 min to obtain the final WPU emulsion.

### 2.3. Characterization

(1) Determination of –NCO Content: The –NCO content of the PU prepolymer was determined by the acetone–dibutylamine titration method. The principle of the method is that –NCO reacts with an excess of dibutylamine in acetone solution, and the remaining dibutylamine is then back-titrated with standardized HCl after complete reaction of –NCO. Approximately 1.0000 g of sample was weighed into a dry iodine flask, dissolved in 10 mL of acetone, and then accurately mixed with 20.00 mL of dibutylamine–acetone solution. The flask was stoppered, shaken thoroughly, and allowed to stand for 15 min. Three drops of bromocresol green–ethanol indicator were added, and the mixture was titrated with 0.1 mol/L HCl standard solution to the endpoint, indicated by a color change from blue to yellow.
(2) Nuclear Magnetic Resonance Spectroscopy: A JNM-ECZ400S/L1 spectrometer (JEOL, Tokyo, Japan) was used for analysis. Samples were dried briefly at 30 °C only to remove volatile components prior to ^1^H NMR analysis. About 10 mg of the dried sample was dissolved in 1.5 mL of deuterated chloroform (CDCl_3_), and an appropriate amount of the resulting solution was transferred into an NMR tube for measurement. The drying step was used solely for sample preparation and was not taken as direct proof of complete structural preservation of the prepolymer. The chemical-shift range was 0–12 ppm.
(3) Fourier-Transform Infrared Spectroscopy (FTIR): The chemical structures of the samples were characterized using a Nicolet iS50 FTIR spectrometer (Thermo Fisher Scientific, Waltham, MA, USA). Before testing, the samples were mixed with AIBN at 0.3 wt% based on resin solids and cast into films in an oven at 130 °C. Unless otherwise stated, the same initiator type and dosage were used in the curing-related FTIR, DSC, and TGA experiments. Spectra were collected in ATR mode at a resolution of 4 cm^−1^ over the range 4000–400 cm^−1^ with 64 scans for each sample.
(4) Differential Scanning Calorimetry (DSC): DSC measurements were carried out on an HCT-1 integrated thermal analyzer (Hengjiu, Beijing, China). Approximately 10–20 mg of dried sample was placed in a crucible and heated from 30 to 200 °C at a rate of 10 °C/min under a nitrogen flow of 100 mL/min, while the heat-flow change during heating was recorded.
(5) Thermogravimetric Analysis (TGA): TGA was performed on an SDT Q600 thermogravimetric analyzer (TA Instruments, New Castle, DE, USA). Samples mixed with thermal initiator were first cast into films and dried in an oven at 130 °C. Approximately 7–10 mg of the resulting film was then placed in a crucible and heated from 30 to 600 °C at 10 °C/min under a nitrogen flow of 100 mL/min to record the weight-loss behavior.
(6) Laser Particle Size Analysis: Particle-size distributions of the emulsions were measured at room temperature with a Malvern Mastersizer 2000 laser particle-size analyzer (Malvern, Worcestershire, UK). Before testing, an appropriate amount of sample was diluted with deionized water and then homogenized at 12,000 r/min for 2 min to obtain a uniformly dispersed sample.
(7) Viscosity and pH Measurements: The viscosity and pH of the WPU emulsions were measured at 25 °C using an NDJ-8S digital rotational viscometer (Youyi, Shanghai, China) (No. 1 spindle, 60 rpm) and a PHS-3C pH meter, respectively. Each sample was measured at least three times in parallel, and the average value was reported.
(8) Contact Angle Analysis: The water contact angle was measured using a DSA30 contact-angle analyzer (KRÜSS, Hamburg, Germany). WPU emulsions were mixed with a fixed amount of thermal initiator, cast into films in an oven at 130 °C, and cut into specimens of approximately 5 mm × 5 mm. Measurements were performed by the static sessile-drop method, and images were collected within 25 s after droplet formation. Each sample was tested seven times, and the average value was used. Wetting behavior was analyzed according to Young’s equation.

$$\cos\theta =\frac{\gamma_{\mathrm{sv}}-\gamma_{\mathrm{sl}}}{\gamma_{\mathrm{lv}}}$$

**Figure 1:**
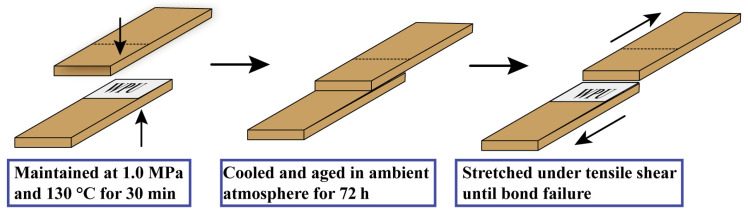
Schematic illustration of moso bamboo bonding and tensile-shear testing.

(9) Mechanical Properties: Tensile tests were carried out at room temperature using an MTS universal testing machine, as shown in Figure 1. Moso bamboo used for bonding was purchased from a bamboo-processing factory in Fujian, China. The bamboo was cut into strips measuring 75 × 25 × 2 mm^3^. Adhesive was applied to a 25 × 25 mm bonding area at a coating weight of 250 g/m^2^, and the bamboo veneers were hot-pressed at 130 °C for 30 min. Tensile shear strength was evaluated under both dry and wet conditions. For dry testing, bonded specimens were cured, dried, and then stored at room temperature for 72 h before measurement. For the boiling-water resistance test, specimens were boiled in water for 4 h, dried in a forced-air oven at (63 ± 3) °C for 20 h, boiled again for 4 h, and then cooled in water at room temperature for 10 min before tensile shear testing. For wet-strength testing, specimens were immersed in water at 63 °C for 3 h before testing. All tests were repeated at least five times, and the average values were reported.

(10) Equilibrium Swelling Analysis: Equilibrium swelling experiments were carried out on the fully cured films. The cured samples were cut into small pieces, dried to constant weight, and immersed in tetrahydrofuran at 25 °C for 24 h. The swollen samples were then removed, gently wiped to remove surface solvent, and weighed immediately. The swelling degree was calculated from the mass change before and after swelling. At least three parallel specimens were tested for each formulation, and the average values were reported.

## 3. Results and Discussion

**Figure 2:**
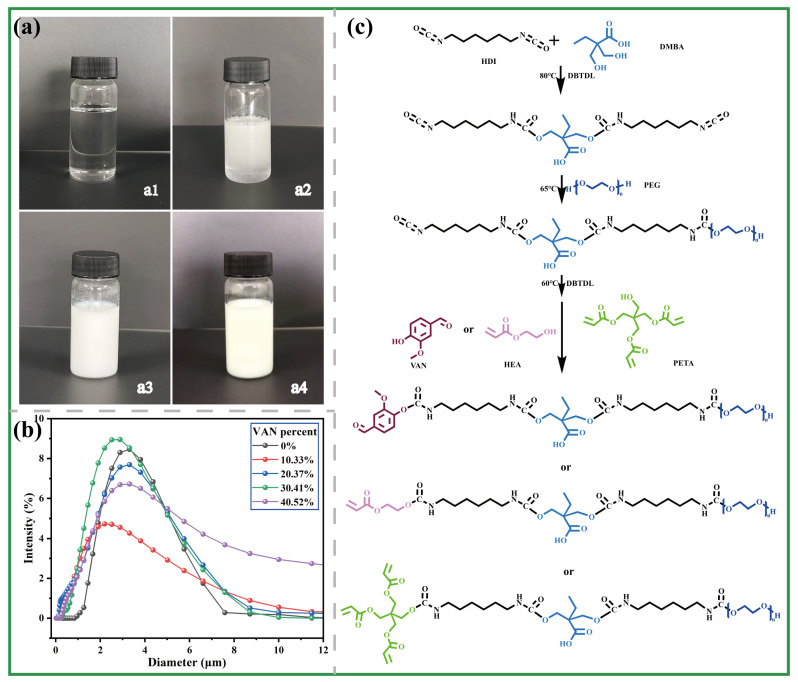
WPU dispersion and design/synthetic strategy. (**a1**) HDI–DMBA hydrophilic center, (**a2**) VAN–HEA/PETA dual-blocked PU prepolymer with a VAN content of 20.37%, (**a3**) fully emulsified WPU dispersion, and (**a4**) WPU dispersion after standing for 7 days; (**b**) particle-size distributions of WPU dispersions with different VAN contents; (**c**) synthetic reaction mechanism of the VAN–HEA/PETA dual-blocked PU prepolymer.

Vanillin contains several key functional groups, including an aldehyde group (–CHO), a phenolic hydroxyl group (–OH), and a methoxy group (–OCH_3_), which endow it with both chemical reactivity and structural tunability. Vanillin contains several key functional groups, including an aldehyde group, a phenolic hydroxyl group, and a methoxy group, which endow it with both structural tunability and potential latent-curing functionality [13]. In the present work, vanillin was introduced as a latent isocyanate-related component, while HEA/PETA served as acrylate-bearing reactive modifiers in the polyurethane system. To achieve stable emulsification under solvent-free conditions, an HDI–DMBA hydrophilic center was first established, after which mixed macropolyols were used for chain extension to form an NCO-terminated polyurethane prepolymer. VAN and the acrylate components were then introduced to regulate terminal-group chemistry and provide additional curing functionality, yielding a dual-blocked PU prepolymer with a milky-white, semitransparent appearance, as shown in Figure 2a1–a4. After neutralization and high-shear self-emulsification, the dual-blocked prepolymer could be stably dispersed in pure water, ultimately affording a milky-white WPU dispersion. By adjusting the proportion of hydrophilic components and controlling the system pH at approximately 6.5 (Figure S1), the resulting dispersion exhibited an average particle size of about 2.5 μm, as shown in Figure 2b. Owing to the solvent-free route and the relatively high viscosity of the modified prepolymer, the particle size was larger than that commonly reported for many conventional WPU dispersions, but the dispersion nevertheless remained stable within the storage period examined in this work.

In most reported WPU studies, organic solvents such as acetone and ethyl acetate are typically required during prepolymer synthesis to control viscosity and enable chain growth, making it still challenging to directly construct a WPU system capable of stable emulsification under solvent-free conditions [6]. In the present work, VAN and the acrylate components were introduced as functionally distinct modifiers of the terminal isocyanate chemistry. During subsequent thermal curing, the VAN-containing structure contributes a higher-temperature latent isocyanate-related reaction, whereas the retained unsaturated groups can undergo free-radical polymerization in the presence of an initiator. A schematic illustration of the mechanism is shown in Figure 2c. This design maintains relatively low viscosity at the prepolymer stage while enabling dual curing-related pathways during thermal treatment. In this system, VAN and HEA/PETA contribute mainly to latent isocyanate-related reaction and unsaturated-bond curing, respectively, endowing the WPU dispersion with staged dual-curing behavior during adhesive curing. This serves as a crucial structural foundation for achieving high adhesive strength and enhanced resistance under wet conditions.

**Figure 3:**
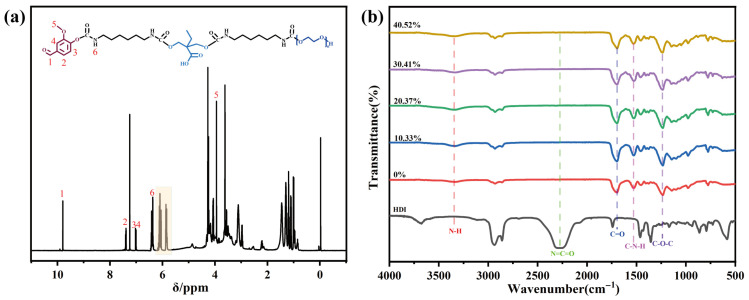
Structural analysis of the VAN–HEA/PETA dual-blocked PU prepolymer. (**a**) ^1^H NMR spectrum of the VAN–HEA/PETA dual-blocked PU prepolymer with a VAN content of 20.37%; (**b**) FTIR spectrum of the product obtained after curing the prepolymer at 130 °C.

The chemical structures of the dual-blocked PU prepolymer and its cured products were characterized by ^1^H NMR and FTIR. Figure 3a shows the ^1^H NMR spectrum of the VAN–HEA/PETA dual-blocked PU prepolymer. A characteristic singlet assigned to the aldehydic proton (–CHO) of vanillin appears at δ 9.81 ppm, together with aromatic and methoxy signals attributable to vanillin-derived units. These signals qualitatively support the presence of vanillin-derived structures in the modified prepolymer. In the HEA/PETA-blocked PU prepolymer prepared without vanillin, these signals are absent, whereas distinct doublet/multiplet signals are observed in the δ 5.8–6.4 ppm region, which can be assigned to protons on the unsaturated double bonds (Figure S2). It should be noted that because of the broad and partially overlapping backbone/polyol resonances in this multicomponent prepolymer system, the ^1^H NMR data were used for qualitative structural assignment rather than for rigorous quantification of VAN incorporation efficiency. The C=C stretching vibration was also observed at 1636 cm^−1^ in the FTIR spectrum of the uncured VAN–HEA/PETA dual-blocked prepolymer (see Figure S3), but this band disappears after the VAN–HEA/PETA dual-blocked PU prepolymer was cured at 130 °C, as shown in Figure 3b. Meanwhile, no obvious characteristic absorption of –NCO was observed near 2270 cm^−1^ in the FTIR spectra, suggesting substantial consumption of terminal isocyanate groups within the detection limit of the method. Quantitative –NCO titration further showed that, for the representative formulation with 20.37% VAN, the blocking efficiency exceeded 99% after the blocking step (Figure S5). In contrast, the broad N–H stretching band near 3300 cm^−1^, the carbonyl absorption near 1696 cm^−1^, the N–H bending vibration near 1530 cm^−1^, and the C–O–C absorption near 1260 cm^−1^ were all clearly present in the FTIR spectrum of the uncured VAN–HEA/PETA dual-blocked prepolymer, confirming the formation of the characteristic urethane structure in the system. As the VAN content increased from 0% to 40.52%, the carbonyl region exhibited progressive broadening, which can be attributed to overlap between the aldehyde carbonyl of VAN and the urethane carbonyl, as well as perturbation of the local hydrogen-bonding environment caused by the aromatic rigidity of VAN and the participation of phenolic hydroxyl groups in hydrogen bonding. Additionally, the appearance of an absorption band corresponding to the aromatic skeleton near 1527 cm^−1^ further indicates the successful incorporation of VAN into the polyurethane network. Together, ^1^H NMR analysis of the prepolymer and FTIR analysis before and after curing confirm that the double-bond structure was retained in the prepolymer prior to curing and underwent free-radical polymerization during the curing process, establishing an intermolecular crosslinked network among the prepolymer chains. Meanwhile, the VAN-blocked isocyanate groups underwent deblocking and subsequent reaction, generating new urethane linkages and further enriching the crosslinked network. Collectively, these results demonstrate that both VAN and HEA/PETA participated in terminal group regulation, providing the structural basis for the dual reactions that occur during subsequent thermal curing.

**Figure 4:**
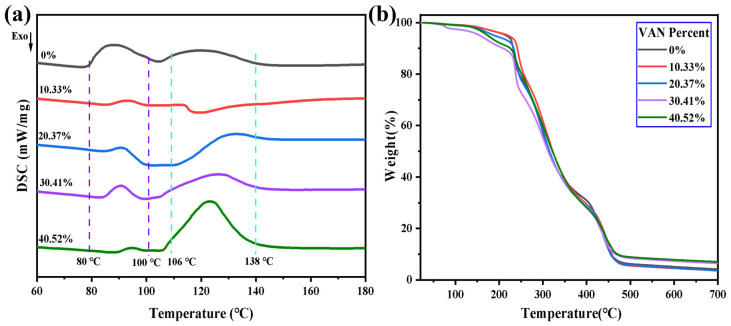
Thermodynamic analysis of the VAN–HEA/PETA modified PU prepolymer. (**a**) DSC curves of PU prepolymers dried at 50 °C; (**b**) TG curves of samples fully cured at 130 °C.

During heating, two latent reactions coexist in the VAN–HEA/PETA dual-blocked PU prepolymer: the VAN-blocked terminal isocyanates undergo deblocking at a certain temperature and subsequently participate in further reactions, while the unsaturated double bonds retained along the prepolymer chains undergo free-radical polymerization in the presence of an initiator. The DSC curves provide evidence for these processes through the evolution of heat flow. As shown in Figure 4a, with increasing VAN content, the DSC curve of the sample gradually evolves from a relatively single heat-flow pattern into a more pronounced two-stage characteristic. In the range of 80–100 °C, all samples exhibit a similar exothermic response, which is mainly associated with AIBN-initiated free-radical polymerization of the unsaturated groups [28,29]. The thermal effect in this region decreases with increasing VAN proportion, consistent with the corresponding reduction in HEA/PETA content in the formulation. In the range of 106–138 °C, a second-stage thermal event appears in all samples, with the corresponding peak becoming progressively stronger and broader as the VAN content increases, indicating that this event is closely related to the VAN-containing structure. Combined with the structural characterization results (Figure 3a), this stage is reasonably associated with thermal activation of vanillin-derived latent isocyanate-related species, with a particularly intense reaction region centered around 120 °C, consistent with literature reports [13,17]. Taken together, these results support dual-curing behavior with two distinguishable thermal events during heating. Figure 4b shows the TGA curves of the cured products. All samples exhibit only minor weight loss below 150 °C, primarily attributable to the volatilization of trace low-molecular-weight residues. A first weight-loss peak appears in the 150–350 °C range, with a maximum weight-loss rate around 238 °C, which can be attributed to cleavage of urethane linkages and partial rupture of weaker bonds. A second weight-loss event appears between 350 and 550 °C, associated with further decomposition or rearrangement of the more stable backbone and char structures. Collectively, these results indicate that the VAN–HEA/PETA dual-blocked PU system exhibits good thermal stability up to 238 °C.

**Figure 5:**
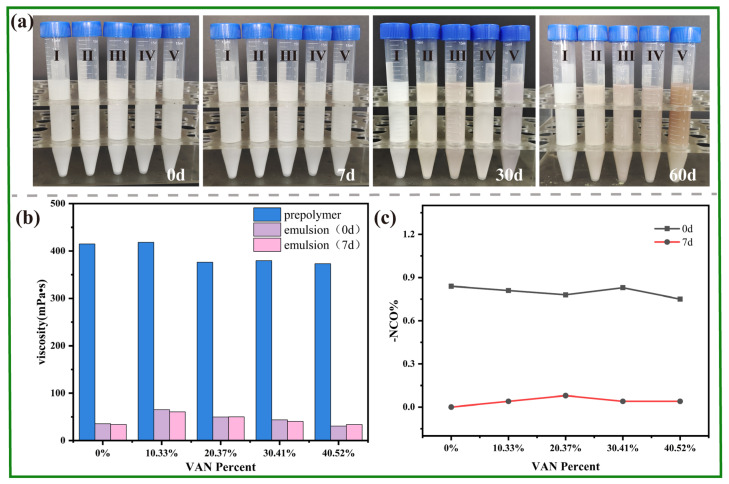
Physicochemical properties and –NCO content variation of the self-emulsified WPU emulsion based on the VAN-HEA/PETA dual-blocked PU prepolymer. (**a**) macroscopic appearance of the emulsion after standing for 0, 7, 30, and 60 days after emulsification, the numbers I–V correspond to emulsions with 0%, 10.33%, 20.37%, 30.41%, and 40.52% VAN content, respectively; (**b**) viscosity of the prepolymer, freshly prepared emulsion, and emulsion after standing for 7 days; (**c**) free –NCO content of the freshly prepared emulsion and the emulsion after standing for 7 days.

The modified PU prepolymer exhibited relatively high viscosity prior to emulsification. TEA was therefore added to partially neutralize the DMBA-derived carboxylic acid groups and reduce intermolecular interactions before water dispersion. Based on the amounts used, the TEA dosage corresponded to 85% neutralization of the acid equivalents. After subsequent addition of dilute KOH solution under high shear, a stable WPU dispersion was obtained. Figure 5a presents the macroscopic appearance of the dispersion after different storage times. During the first 30 days, the dispersions remained macroscopically stable without obvious phase separation, although VAN-containing samples showed gradual color deepening. At longer storage times, precipitation appeared in samples with higher VAN contents (>30.41%), indicating that storage stability remained composition-dependent. The WPU emulsions exhibited a much lower viscosity than the corresponding prepolymer, with an average value of approximately 50 mPa·s (Figure 5b). Only minor changes in viscosity were observed during storage, indicating good colloidal stability. Figure 5c presents the results for free –NCO content in the freshly prepared emulsion and after standing for 7 days. A small amount of residual free –NCO (approximately 0.8%) was still detected in the freshly prepared emulsion. This residual –NCO is attributed mainly to the intentionally sub-stoichiometric blocking design (blocking component/residual –NCO = 0.9:1) together with incomplete terminal-group consumption under practical reaction conditions, rather than to substantial deblocking during emulsification. During storage, the residual free –NCO was gradually consumed, indicating that limited post-emulsification evolution of the dispersion still occurred. Combined with the viscosity results (Figure 5b), these observations indicate that the PU prepolymer maintained a relatively stable latent structure at room temperature, thereby ensuring dispersion stability.

**Figure 6:**
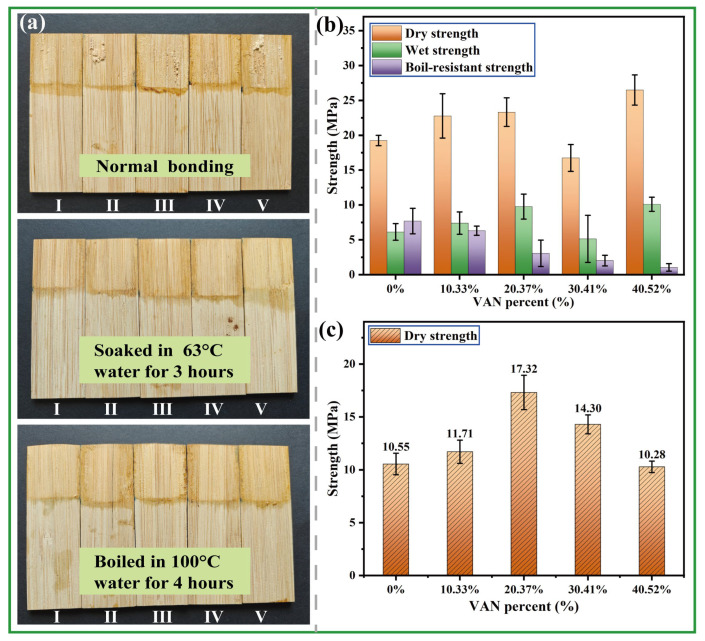
Adhesive performance of the WPU emulsion. (**a**) Failure appearance of the bonded bamboo surfaces after hot bonding at 130 °C followed by storage at room temperature, immersion in water at 63 °C, and boiling-water treatment at 100 °C. The numbers I–V correspond to bamboo samples bonded with emulsion containing 0%, 10.33%, 20.37%, 30.41%, and 40.52% VAN content, respectively; (**b**) tensile shear strength corresponding to the failure modes in (**a**); (**c**) dry bonding strength of the WPU emulsion after standing for 30 days.

Moso bamboo, with its dense surface, limited wettability, and low interfacial reactivity, is a relatively difficult substrate to bond. It was therefore selected as the adherend to evaluate the practical adhesive performance of the prepared WPU emulsion via hot pressing. The tensile-shear performance of the bonded specimens was then evaluated under three conditions: dry state at room temperature, after high-humidity treatment, and after boiling-water treatment. Figure 6a shows the failure patterns under different test conditions, and the corresponding tensile-shear strengths are presented in Figure 6b. For dry specimens without water treatment, obvious bamboo failure appeared on the fractured surfaces once the VAN content exceeded 10.33%, indicating that the adhesive-layer strength had approached or exceeded the local intrinsic strength of the bamboo substrate. Quantitatively, when the VAN content was below 30.41%, the dry strength first increased and then decreased with increasing VAN content, reaching an optimum value above 23 MPa at 20.37%; when the VAN content was increased further, the dry strength exhibited a partial recovery. Wet strength followed a similar trend and also reached a relatively high value of 9.5 MPa at a VAN content of 20.37%. To further probe the network structure of the cured adhesive layers, equilibrium swelling experiments were carried out. The swelling degrees of the samples with VAN contents of 0%, 10.33%, 20.37%, 30.41%, and 40.52% were 92.62%, 93.30%, 96.79%, 89.65%, and 91.75%, respectively (Table S1 and Figure S7). Under identical swelling conditions, a lower swelling degree generally corresponds to a denser effective network. Accordingly, the effective network density changed in a non-monotonic manner with VAN content and reached its highest level at 30.41% VAN. Notably, this trend did not coincide with that of bonding strength, which peaked at 20.37% VAN. This result indicates that the adhesive performance of the present system was not governed solely by network density. Rather, the optimum bonding performance at 20.37% VAN is attributed to a more favorable balance among crosslinking level, network rigidity, interfacial wetting, and stress dissipation within the adhesive layer. In contrast to the dry- and wet-strength results, the boiling-water strength decreased from 7.5 MPa to 1.2 MPa with increasing VAN content, indicating that the interfacial stability of the system remained inadequate under severe hydrothermal conditions. Contact-angle measurements confirmed that the WPU emulsions exhibited good wetting ability, which improved with increasing VAN content (Figure S4). At the same time, the introduction of VAN increased the polarity and water sensitivity of the cured system, thereby facilitating water penetration under boiling-water conditions. The resulting water uptake could plasticize the adhesive layer, weaken intermolecular interactions, and accelerate deterioration of the adhesive/substrate interface [30,31,32]. Figure 6c shows that the dry bonding strength of emulsions stored for 30 days was generally lower than that of freshly prepared samples. Particle-size analysis after 30 days of storage (Figure S6) showed a marked increase in particle size, with the average particle size reaching about 8 μm, indicating gradual physical aging of the emulsion accompanied by particle growth and likely partial coalescence. Such changes could reduce the uniformity of film formation and the efficiency of subsequent curing, thereby lowering the final bonding strength. Prolonged storage may also decrease the effectiveness of the unsaturated curing sites because of gradual microstructural evolution and reduced chain mobility within the emulsion particles.

## 4. Conclusions

By integrating vanillin-derived latent isocyanate-related structures with acrylate-bearing reactive modifiers into a self-emulsifying polyurethane system, this work established a solvent-free WPU dispersion with dual-curing characteristics under thermal treatment. Structural characterization confirmed the introduction of vanillin-derived units into the prepolymer and the retention of polymerizable unsaturated groups before curing, while thermal analysis revealed two distinguishable curing-related thermal events, reflecting a staged dual-curing process within the studied system. Within the investigated composition range, the formulation containing 20.37% VAN achieved the most favorable balance of performance, delivering high dry and wet shear strengths together with stable dispersion behavior, thereby demonstrating that the present vanillin/acrylate design is an effective way to couple latent reactivity, curing control, and practical adhesive performance in a solvent-free WPU platform. Meanwhile, the present study also identifies the main limitations of the current system, including reduced boiling-water resistance at higher VAN contents, color deepening during storage, and loss of dry bonding strength after prolonged emulsion storage. These findings indicate that the vanillin/acrylate strategy is promising but not yet fully optimized for long-term hydrothermal service. Nevertheless, the present work establishes a useful dual-curing design concept for solvent-free WPU adhesives and provides a meaningful basis for further development of high-performance latent-curing waterborne polyurethane systems.

## Figures and Tables

**Table 1:** Molar formulation of the WPU prepolymers with different VAN contents.

	VAN Percent (%)	0%	10.33%	20.37%	30.41%	40.52%
Chemical Reagent (mol)						
PCDL		0.040	0.040	0.040	0.040	0.040
PPG		0.042	0.042	0.042	0.042	0.042
PEG		0.018	0.018	0.018	0.018	0.018
DMBA		0.053	0.053	0.053	0.053	0.053
HDI		0.300	0.300	0.300	0.300	0.300
VAN		0.0	0.026	0.051	0.077	0.102
HEA		0.205	0.184	0.164	0.100	0.123
PETA		0.051	0.046	0.041	0.036	0.031
TEA		0.046	0.046	0.046	0.046	0.046
KOH		0.007	0.007	0.007	0.007	0.007

Note: VAN, HEA, and PETA were premixed before addition. The total molar feed ratio of the VAN/HEA/PETA mixture to residual –NCO groups was fixed at 0.9:1 for all formulations.

## Data Availability

The original contributions presented in this study are included in the article/Supplementary Materials. Further inquiries can be directed to the corresponding authors.

## Supplementary Materials

The following supporting information can be downloaded at: https://www.mdpi.com/article/10.3390/polym18080975/s1, Figure S1: pH variation curve of the VAN–HEA/PETA dual-blocked PU prepolymer with a VAN content of 20.37% as a function of increasing TEA content; Figure S2: ^1^H NMR spectrum of the HEA/PETA-blocked PU prepolymer; Figure S3: FTIR spectra of the VAN–HEA/PETA dual-blocked PU prepolymer before curing: (a) full spectrum and (b) magnified region from 2000 to 1200 cm^−1^;. Figure S4: Contact angles of WPU emulsions with different VAN contents. Figure S5: Comparison of –NCO contents before and after the blocking step for the formulation with 20.37% VAN; Figure S6: Particle-size analysis after 30 days of storage; Table S1: Swelling degrees of samples with different VAN contents; Figure S7: Samples with different VAN contents before and after soaking in tetrahydrofuran for 24 h.
