# Supplementary material for: Solvent-Free Dual-Curable Waterborne Polyurethane Adhesives Based on Vanillin and Acrylate Monomers
Source: Polymers (Basel). 2026 Apr 17;18(8):975. doi: 10.3390/polym18080975 (PMC13120263; doi:10.3390/polym18080975)
Supplement: Supplementary file 1 [file polymers-18-00975-s001.zip › polymers-4243728-supplementary.pdf]

Solvent-Free Dual-Curable Waterborne Polyurethane Adhesives Based on Vanillin and Acrylate Monomers

Weiling Hu [1,2], Xiao Zhang [2], Hao Li [2], Hengyuan Liang [2], Can Lin [2], Zhuo Li [2], Jia Liu [2,*], Feng Feng [1,*]

1 State Key Laboratory of Green Chemical Synthesis and Conversion, Zhejiang Key Laboratory of Surface and Interface Science and Engineering for Catalysts, Zhejiang University of Technology, Hangzhou 310014, China

2 College of Chemical and Material Engineering, Quzhou University, Quzhou 324000, China

* Correspondence: liujia1@qzc.edu.cn (J. L.); ffeng@zjut.edu.cn (F. F.)

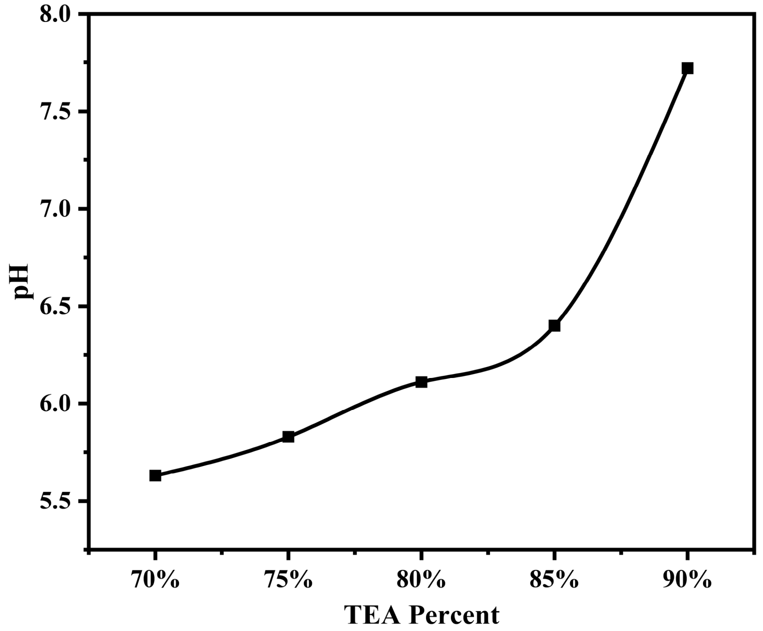

Figure S1. pH variation curve of the VAN–HEA/PETA modified PU prepolymer with a VAN content of 20.37% as a function of increasing TEA content.

To achieve efficient self-emulsification, triethylamine (TEA) was introduced after prepolymer synthesis to ionize the carboxylic acid groups and weaken interchain hydrogen bonding, thereby reducing internal friction. As shown in Fig. S1, when the TEA loading approached 80% of the carboxylic acid molar content, the pH of the prepolymer reached approximately 6.2. Under these conditions, rapid emulsification was achieved by adding a weakly alkaline KOH solution under high-shear mixing. The best emulsion stability was obtained at a final pH of around 6.5, whereas pH values above 7 or below 4 led to sedimentation upon standing.

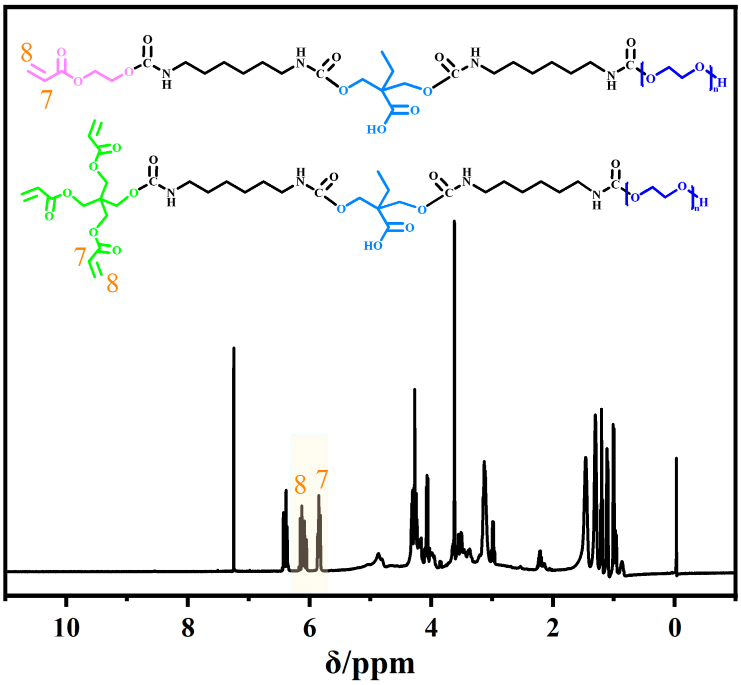

Figure S2. ¹H NMR spectrum of the VAN–HEA/PETA modified PU prepolymer.

The ¹H NMR spectrum (400 MHz, $CDCl_3$) of the HEA/PETA-blocked PU prepolymer is presented in Fig. S2. Intense multiplets in the range of δ 6.46–5.83 are assigned to the vinyl protons (–CH=CH_2) derived from the HEA/PETA acrylate-bearing components, with complex splitting patterns reflecting the microenvironment of the polymer chains. Signals in the region of δ 4.29–3.57 correspond to oxymethylene (–OCH_2–) and methoxy (–OCH_3) protons from the polyether soft segments and acrylate side chains. The strong singlet at δ 3.13 (28H) and doublet at δ 2.98 (J = 7.3 Hz, 4H) are attributed to methylene groups adjacent to nitrogen or carbonyl functionalities, likely arising from the neutralizing agent (e.g., triethylamine) and the soft-segment structures of the polyurethane prepolymer, respectively. The aliphatic region (δ 1.47–1.01) displays multiple intense signals corresponding to methyl and methylene protons from the polyurethane backbone, including characteristic resonances of the hexamethylene units derived from HDI.

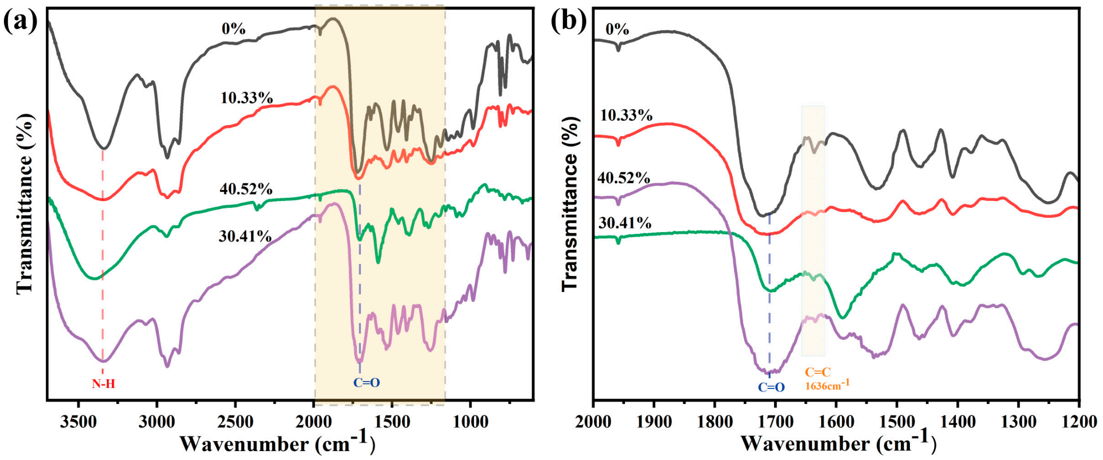

Figure S3. FTIR spectra of the VAN–HEA/PETA modified PU prepolymer before curing: (a) full spectrum and (b) magnified region from 2000 to 1200 cm^{-1}.

Figure S3 shows the FTIR spectra of the VAN–HEA/PETA dual-blocked PU prepolymer before curing. For samples with different VAN contents, no characteristic –NCO absorption was observed at 2270 cm^{-1}, suggesting substantial consumption of terminal isocyanate groups within the detection limit of FTIR. The corresponding blocking efficiency was further supported by quantitative –NCO titration, as shown in Fig. S5. Typical urethane absorptions—including the broad N–H stretching band near 3300 cm^{-1} and the carbonyl absorption near 1696 cm^{-1}—were clearly present. In the magnified spectrum (Fig. S3b), the C=C stretching vibration absorption can be observed at 1636 cm^{-1}.

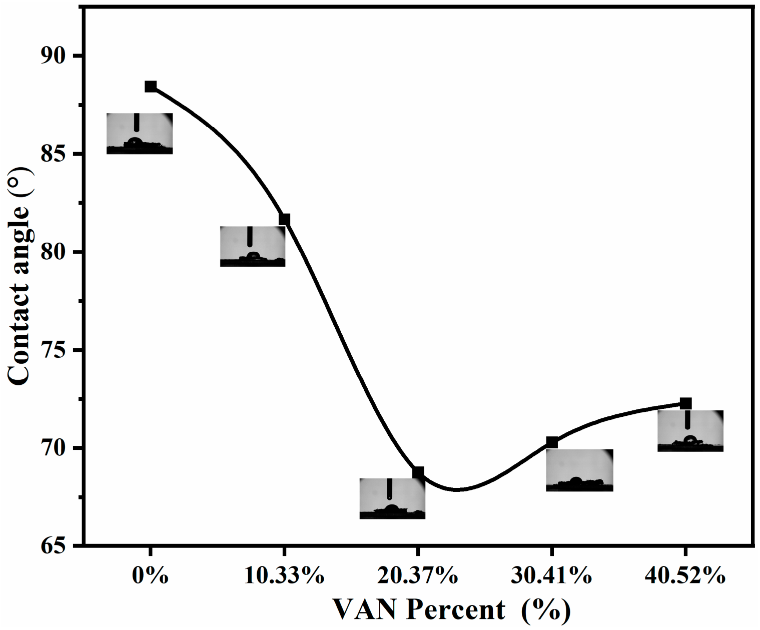

Figure S4. Contact angles of WPU films with different VAN contents.

Contact angle measurements were used to evaluate the surface wettability of the cured WPU films. The WPU emulsions were mixed with a thermal initiator and cured into films at 130 °C, after which static sessile drop measurements were performed. Static sessile drop measurements were then performed, and the results are shown in Fig. S4. At a VAN content of 0, the water contact angle was approximately 88°. As the VAN content increased, the contact angle gradually decreased, reaching a minimum of about 68° at 20.37% VAN, indicating the highest water wettability among the investigated formulations. At higher VAN contents, the contact angle increased slightly again, indicating that the effect of VAN on surface wettability was also non-monotonic.

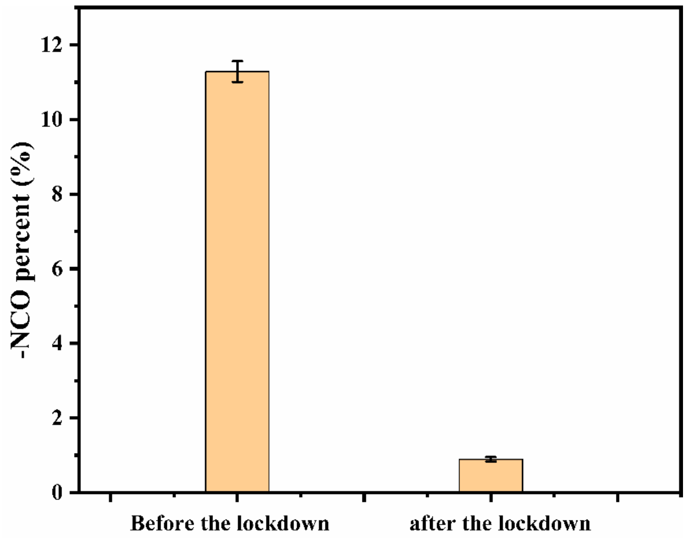

Figure S5. Comparison of –NCO contents before and after the blocking step for the formulation with 20.37% VAN.

For the representative formulation with 20.37% VAN, the average –NCO content decreased from 11.286% before the blocking step to 0.891% after blocking. The theoretical and measured –NCO consumptions were 10.434% and 10.395%, respectively, corresponding to a blocking efficiency above 99%. These results indicate that the blocking step proceeded efficiently under the applied conditions.

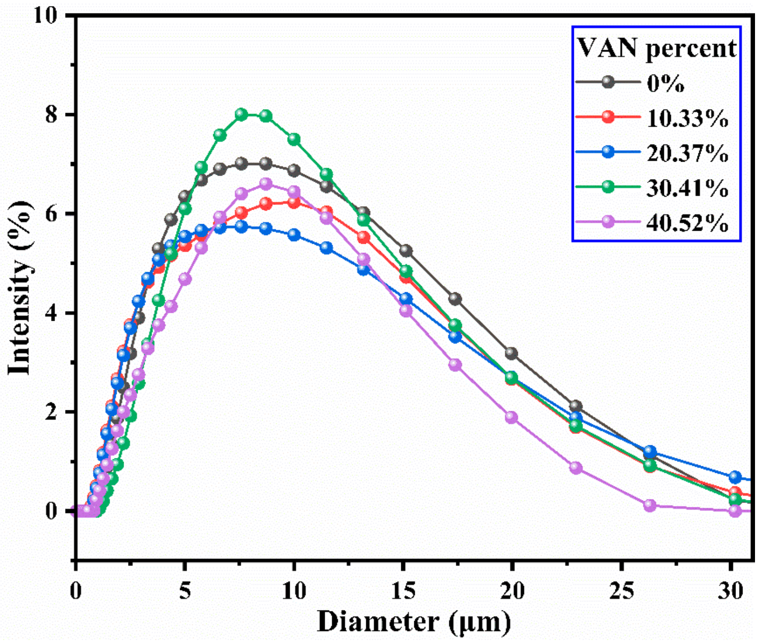

Figure S6. Particle-size analysis after 30 days of storage.

After 30 days of storage, the particle size of the emulsions increased markedly, with the average particle size reaching approximately 8 μm. This result indicates gradual physical aging of the emulsion during storage, accompanied by particle growth and likely partial coalescence, which is consistent with the decrease in bonding strength observed after storage.

Table S1. Swelling degrees of cured samples with different VAN contents.

VAN percent%	0%	10.33%	20.37%	30.41%	40.52%
Swelling degrees	92.62%	93.30%	96.79%	89.65%	91.75%

Under identical swelling conditions, a lower swelling degree indicates a denser effective network. The results show that the effective network density changed non-monotonically with VAN content and reached its highest level at 30.41% VAN. Notably, this trend did not coincide with the variation in bonding strength, indicating that the adhesive performance of the present system was governed not only by network density, but also by the balance among network rigidity, interfacial wetting, and stress dissipation.

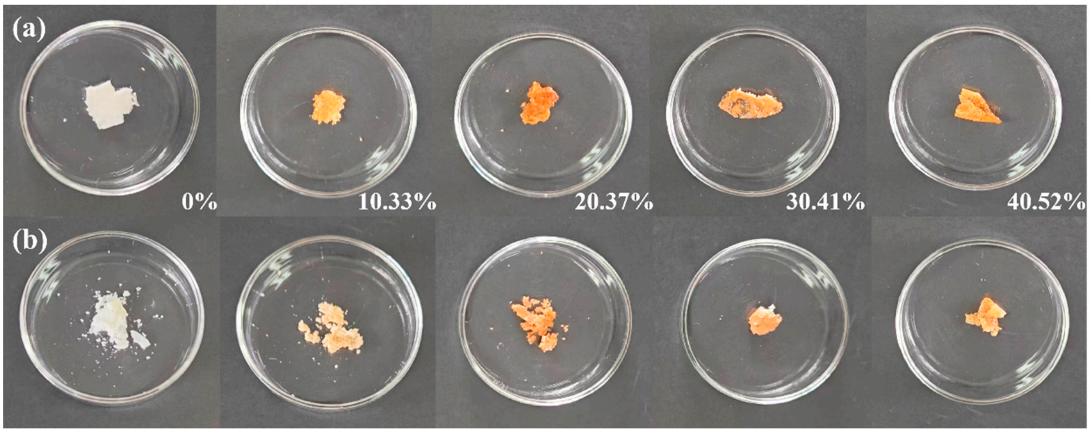

Figure S7. Samples with different VAN contents: (a) samples without tetrahydrofuran soaking, and (b) samples after soaking in tetrahydrofuran for 24 h.

Figure S7 compares the appearance of the cured samples before and after soaking in tetrahydrofuran for 24 h. The retained integrity of the swollen samples further indicates the formation of cured network structures, although their swelling degrees remained relatively high.
